# A Multiparametric Approach Based on NT-proBNP, ST2, and Galectin3 for Stratifying One Year Prognosis of Chronic Heart Failure Outpatients

**DOI:** 10.3390/jcdd4030009

**Published:** 2017-07-15

**Authors:** Dario Grande, Marta Leone, Caterina Rizzo, Paola Terlizzese, Giuseppe Parisi, Margherita Ilaria Gioia, Tiziana Leopizzi, Antonio Segreto, Piero Guida, Roberta Romito, Marco Matteo Ciccone, Francesca Di Serio, Massimo Iacoviello

**Affiliations:** 1 School of Cardiology, University of Bari, Piazza Giulio Cesare 11, 70124 Bari, Italy; dario.grande@ymail.com (D.G.); k.rizzo@live.it (C.R.); paola.terlizzese@yahoo.it (P.T.); giuseppeparisi88@libero.it (G.P.); m.ilariagioia@gmail.com (M.I.G.); tiziana.leopizzi@hotmail.it (T.L.); antonio.segreto1988@gmail.com (A.S.); marcomatteo.ciccone@uniba.it (M.M.C.); 2 Cardiology Unit, S.S. Annunziata Hospital, Via Bruno Francesco 1, 74123 Taranto, Italy; martaleo84@yahoo.it; 3 Scientific Clinical Institutes Maugeri, I.R.C.C.S., Institute of Cassano delle Murge, Contrada Frà Diavolo 13, 70020 Cassano delle Murge, Italy; pieroguida@libero.it; 4 Emergency Cardiology Unit, Policlinic University Hospital, Piazza Giulio Cesare 11, 70124 Bari, Italy; robertaromito@libero.it; 5 Clinic Pathology Unit, Policlinic University Hospital, Piazza Giulio Cesare 11, 70124 Bari, Italy; diseriofrancesca@tiscali.it; 6 Cardiology Unit, Cardiothoracic Department, Policlinic University Hospital, Piazza Giulio Cesare 11, 70124 Bari, Italy

**Keywords:** heart failure, prognosis, biomarkers

## Abstract

Galectin-3 and ST2 are emerging biomarkers involved in myocardial fibrosis. We evaluate the relevance of a multiparametric biomarker approach based on increased serum levels of NT-proBNP, galectin-3, and ST2 in stratifying the prognosis of chronic heart failure (CHF) outpatients. In 315 CHF outpatients in stable clinical condition clinical and echocardiographic evaluations were performed. Routine chemistry and serum levels of NT-proBNP, galectin-3, and ST2 were also assessed. During a 12 month follow-up, cardiovascular death, and/or heart failure (HF) occurred in 64 patients. The presence of NT-proBNP, galectin-3, and ST2 were higher than the recommended cutoffs and were all associated with events at univariate Cox regression analysis, as well as in a multivariate analysis including the three biomarkers. When a score based on the number of biomarkers above the recommended cut-offs was used (in a range of 0–3), it was associated with events both with respect to the univariate (HR 2.96, 95% CI 2.21–3.95, *p <* 0.001, C-index 0.78) and the multivariate (HR 1.52, 95% CI 1.06–2.17, *p:* 0.023, C-index 0.87) analyses, after correction for the variables of a reference model. Our results suggest that an easy prognostic approach based on the combination of three biomarkers, although with partially-overlapping pathophysiological mechanisms, is able to identify patients with the highest risk of heart failure progression.

## 1. Introduction

Over the last few decades there has been a growing interest in the use of biomarkers for diagnosis, prognostic stratification, and guiding therapy in heart failure (HF) patients [1]. Natriuretic peptides are certainly those whose clinical usefulness in daily clinical practice is supported by evidence. However, emerging biomarkers are being studied in order to overcome the limits of natriuretic peptides linked to confounding factors, such as age, atrial fibrillation, obesity, and renal function. Among those, galectin-3 (Gal-3) and suppression of tumorigenicity-2 (ST2) seem to be particularly promising. Both these novel biomarkers reflect different pathophysiological pathways compared with natriuretic peptides, i.e., cardiac remodeling related to fibrosis. Moreover, Gal-3 also seems associated to renal dysfunction [2,3].

Considering the distinct information carried by these three biomarkers, they could be used in order to achieve an improvement in the predictive accuracy of events. However, there are conflicting results regarding this issue. Several studies, including a recent meta-analysis [4], investigated the outcome prediction of Gal-3 in chronic heart failure, being generally considered a significant predictor for cardiac events even after adjusting for natriuretic peptide levels [5,6,7,8]. Nevertheless, in other series, this association lost its significance after adjustment for NT-proBNP [9,10] or glomerular filtration rate (GFR) [11]. On the other hand, ST2 has shown to be a significant prognostic marker for chronic heart failure (CHF) patients [12,13], although in some cohorts of patients it was associated with outcomes, but did not significantly affect reclassification of risk [14,15]. However, most of the studies testing the role of the new biomarkers were retrospective and involved study populations of clinical trials which present different clinical characteristics compared to a real world population.

Finally, few studies were aimed at prospectively evaluating the possible usefulness of a multi-biomarker approach in order to stratify short- and mid-term prognosis and support their possible usefulness for differentiating patients’ risk and better planning follow-ups.

The aim of our study was to investigate the possible integration of information carried by increased serum levels of NT-proBNP, Gal-3, and ST2 in order to better stratify the risk for heart failure progression in a group of chronic heart failure outpatients.

## 2. Materials and Methods

A population of consecutive outpatients affected by CHF of any origin was referred to the Heart Failure Unit of the Cardiology Unit of Policlinic Hospital of Bari and was enrolled between May 2013 and May 2016. At the time of enrollment, they had been clinically stable for at least 30 days (i.e., no significant changes in hemodynamic status and in medical therapy) and had received conventional medical and electrical therapy for at least three months previously, if not contraindicated. Patients with acute decompensated heart failure (ADHF) and renal failure requiring dialysis were excluded from the study. The study was approved by the local ethics committee and all patients gave their written informed consent for the study.

*Baseline evaluations*. At the time of enrolment all patients underwent a medical visit, an ECG, an echocardiographic examination, and gave a blood sample for chemical evaluations.

*Medical examination and electrocardiogram.* A documented record was made for each patient, including ischemic heart disease, arterial hypertension and diabetes mellitus diagnosis, as well as a history of ventricular arrhythmic events. During medical examination, New York Heart Association (NYHA) class and arterial pressure were evaluated. Heart rhythm and heart rate were assessed by a12-lead ECG.

*Echocardiographic evaluation.* All patients underwent an echocardiographic evaluation using an echocardiograph (Vivid 7, GE Vingmed Ultrasound, General Electric, Milwaukee, WI, USA). Left Ventricular end-diastolic (LVEDV) and end-systolic (LVESV) were recorded as absolute values and adjusted for body surface area, and left ventricular ejection fraction (LVEF) was calculated using Simpson’s rule. Mitral (MR) and tricuspid regurgitation (TR) were semi-quantitatively evaluated with arbitrary units ranging from 0 to 4. Tricuspid annular plane excursion (TAPSE) was used to assess the right ventricular systolic function [16]. Dilatation of the inferior vena cava and its collapsibility during inspiration were evaluated in order to assess the central venous pressure (CVP). CVP was estimated to be equal to 3 mmHg (range 0–5 mmHg) in the presence of a cava vein of normal diameter and good collapse, 15 mmHg in the presence of a dilated cava vein and poor collapse, and 8 mmHg in intermediate cases [16]. Pulmonary artery systolic pressure (PASP) was evaluated on the basis of the peak velocity of TR and an estimation of the CVP. By mitral pulsed Doppler at the level of the mitral leaflets, the peak of the E wave (E) and, by tissue Doppler imaging, the early diastolic velocity peak (E’) at the level of septal and lateral mitral annulus were measured. Then the E/E’ ratio was calculated as recommended.

*Chemical evaluations.* Blood samples were taken, after subjects remained in supine position for at least 10 minutes, in order to evaluate serum creatinine, urea, hemoglobin, NT-proBNP. The GFR was calculated using the Chronic Kidney Disease Epidemiology Collaboration equation (GFRCKD-EPI). Galectin-3 levels were evaluated in the plasma with an enzyme-linked immunosorbent assay (Biomerieux, Marcy-l’Etoile, France), while ST2 was assessed by a kit (Critical Diagnostics, San Diego, CA, USA). In order to assess the presence of abnormal values of NT-proBNP, ST2, and Galectin-3, the current recommended cut-offs were considered (NT-proBNP > 1000 pg/mL [17], ST2 > 34 ng/mL [18], and galectin-3 > 17.8 ng/mL [19]).

*Follow-up.* The patients received follow-up as outpatients according to the protocol of our Heart Failure Unit. The occurrence of death and hospitalizations within the first 12 months were recorded reflecting the underlying cause. Deaths were classified as being due to cardiovascular or non-cardiovascular causes. In cases of death outside the hospital, or in other centers, relatives were interviewed and related medical documentation was acquired. The primary end-point was a composite of cardiovascular death and HF related hospitalization.

*Statistical analysis.* Continuous variables are expressed as mean values ± SD. Categorical variables are expressed as the absolute frequency or percentage. Pearson's linear correlations were used to assess the relationship among biomarkers and the other studied variable.

Comparisons among groups were performed by ANOVA analysis and post-hoc comparisons by Newman-Keuls test comparisons among groups of the categorical variables that were performed with the chi-square test or Fisher test.

The event-free survival curves were based on Kaplan–Meier analyses. Univariate Cox’s proportional hazards model was used to assess the association of variables with the endpoints. For all variables, hazard ratios (HRs) with their 95% confidence intervals (CIs) are given. The final reference model for the primary endpoint was developed with a Cox multivariate regression analysis including all of the univariate predictors by using a forward stepwise selection approach with *p <* 0.05 to retain covariates in the model. To avoid multicollinearity, redundant variables were dropped from the multivariable regression models in the case of pairwise correlations between continuous variables exceeding 0.50 as Pearson’s coefficient, including the variable with the strongest individual effect size [20]. To test the independent association of biomarker scores with primary endpoint, they were analyzed in a Cox multivariate regression analysis including all of the variables of the reference model.

Model discrimination was examined with the c-statistic. To quantify the clinical impact of RRI on the models, integrated discrimination improvement (IDI), and category-free net reclassification improvement (NRI) were calculated. IDI considers the change in the estimated prediction probabilities as a continuous variable. NRI were calculated as the observed event rate increase among the individuals for whom the predicted risk goes up and the observed event rate decrease among those for whom the predicted risk declines [21].

Finally, the independent association of biomarker score with primary end-point was tested by a multivariate regression models separately, including the presence of traditional biomarkers according to the recommended cut-offs (i.e., GFR < 60 mL/min/1.73m^2^, Urea > 50 mg/dL, hemoglobin < 12 g/dL, natremia < 135 mEq/L), the BIOSTAT-CHF [22], and the MAGGIC models [23].

The analyses were carried out using Statistica software, version 6.1 (StatSoft Inc., Tulsa, OK, USA) and STATA software, Version 12 (StataCorp, College Station, TX, USA). *p*-values of <0.05 were considered statistically significant.

## 3. Results

A total of 315 patients were enrolled, 230 (73%) affected by heart failure with reduced ejection fraction, 69 (22%) by heart failure with mid-range ejection fraction and 16 (5%) by heart failure with preserved ejection fraction. Baseline clinical characteristics of study population are shown in Table 1. During the 12 month follow-up the following events were observed: 19 deaths (17 due to cardiovascular causes), and 62 hospitalizations due to worsening HF. The primary endpoint, i.e., cardiovascular death and/or HF related hospitalization, occurred in 64 patients.

### 3.1. Correlates of Biomarkers

As shown in Table 2 NT-proBNP was significantly and negatively correlated with the left ventricular ejection fraction (LVEF), TAPSE, hemoglobin, and GFR-EPI; it was positively and significantly correlated with age, NYHA class, left ventricular end-systolic volume (LVESV), E/e’ ratio, mitral regurgitation (MR) severity, tricuspid regurgitation (TR) severity, Pulmonary artery systolic pressure (PAPS), and central venous pressure (CVP). No correlations were found with body mass index (BMI). ST2 was significantly and negatively correlated with BMI, LVEF, TAPSE, hemoglobin and GFR-EPI; it was positively and significantly correlated with NYHA class, MR, TR, PAPS, and CVP. No correlations were found with age, LVESV, and E/e’. Gal-3 was significantly and negatively correlated with LVEF, TAPSE, hemoglobin, and GFR-EPI; it was positively and significantly correlated with age, NYHA class, E/e’, MR, TR, PAPS, and CVP. No correlations were found with BMI and LVESV.

### 3.2. Independent and Significant Association of Biomarkers with Events

As shown in Table 3, in the univariate regression analysis NT-proBNP > 1000 pg/mL, as well as ST2 > 34 ng/mL and Gal-3 > 17.8 ng/mL were all associated with events. In a multivariate model including the three biomarkers, all remained associated with events. The addition to the presence of NT-proBNP > 1000 pg/mL of ST2 > 34 ng/mL and Gal-3 > 17.8 ng/mL significantly improved discrimination and reclassification (IDI 0.080, *p <* 0.001; free NRI 0.338; *p <* 0.001).

**Figure 1:**
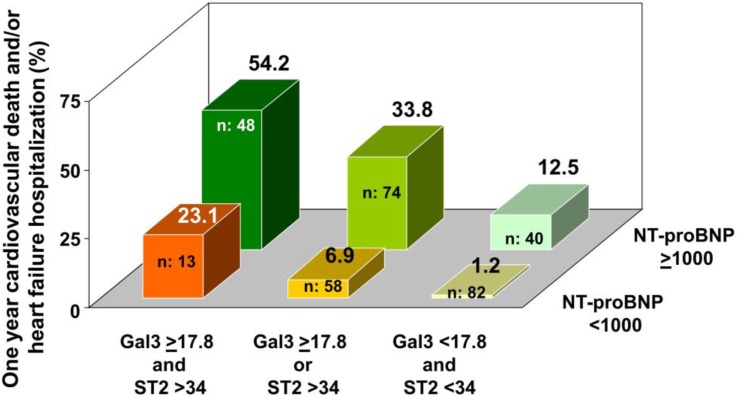
The probability of one-year events according to the combination of the presence of increased NT-proBNP and increased ST2 and/or Gal-3 is shown.

Figure 1 shows the probability of one-year events accordingly to the combination of the presence of an increased NT-proBNP and an increased ST2 and/or Gal-3. Patients with two and three elevated biomarkers showed the greatest risk.

**Figure 2:**
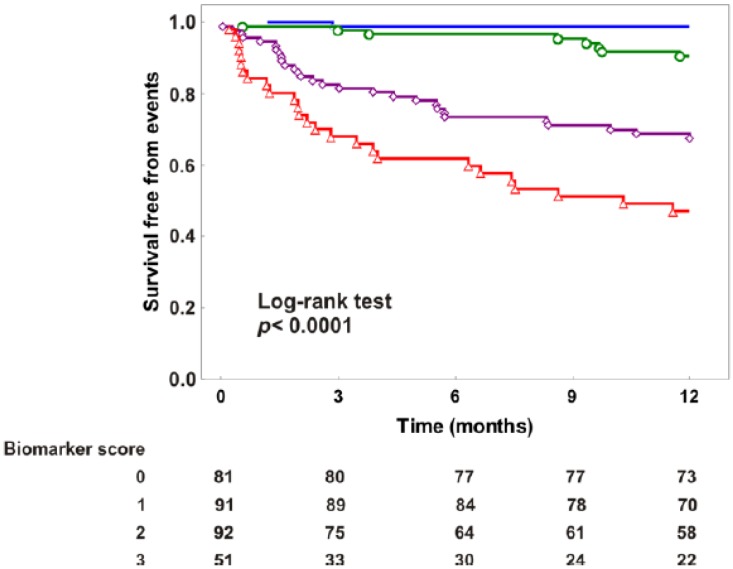
Kaplan-Meier curves according to the number of altered biomarkers are shown.

In Table 1 clinical characteristics of patients according to the number of elevated biomarkers are shown. As shown in the Table 4, the variable computed on the basis on the number of increased biomarkers (range 0–3) was associated with events with respect to the univariate, as well as at the multivariate, analysis, after correction for the variables of a reference model (i.e., failure to prescribe beta blockers, hemoglobin < 12 g/dL, LVEF < 30%, NYHA class 3, at least moderate tricuspid regurgitation). Moreover, when the biomarkers score was added to the reference model, a significant IDI and free NRI were observed. Figure 2 shows Kaplan-Meier curves according to biomarkers score.

Biomarker scores also remained significantly associated with events with respect to the multivariate regression analysis in a model including the traditional biomarkers, i.e., the presence of low sodium levels, of low hemoglobin, high urea, and of a GFR below 60 mL/min with a significant IDI and free NRI (Table 4).

Finally, with respect to the univariate analysis, the biomarker scores showed a C-index greater than that of the BIOSTAT-CHF model (C-index 0.70) and similar to that of the MAGIC score (C-index 0.80). Moreover, an independent and significant association with events was found with respect to the multivariate analysis after correction for BIOSTAT-CHF, as well as after correction with MAGIC score (Table 4). When the biomarker score was added to BIOSTAT-CHF and MAGIC-HF, a significant IDI and a significant free NRI were also observed.

## 4. Discussion

The main findings of this study are that a score based on the integration of three biomarkers, i.e., NT-proBNP, Gal-3, and ST2, provides better prognostic stratification for predicting short and mid-term occurrence of heart failure progression, thus suggesting the possible usefulness of this simple approach in clinical practice.

Risk stratification of patients affected by heart failure is based on a number of clinical and laboratory evaluations. Among these parameters, blood levels of cardiac biomarkers give the possibility of predicting outcomes, in terms of recurrent heart failure-related readmissions and death. Although biomarkers cannot replace the clinical assessment based on history, or physical and laboratory examination, in any case, they can offer an objective and reproducible parameter for risk estimation. This could be useful in those clinical settings, such as primary care and community care medicine, in which cardiological examinations like echocardiograms are not easy to perform.

Up to now the only biomarkers strongly recommended in risk stratification have been natriuretic peptides. They have an established role in the prognostic evaluation of heart failure patients, with the highest grade of recommendation in the last ACCF/AHA HF guidelines for estimating prognosis or disease severity both in the acute and chronic patients [24]. However, there are some limitations to natriuretic peptide interpretation which can impair diagnostic specificity because of a number of confounding factors [25]. Moreover, outcome predictability may vary with the cut-off used. Defining a precise cut-off can be challenging as multiple factors influence blood values, i.e., increasing age, kidney disease, atrial fibrillation, anemia, and sepsis [26,27]. NT-proBNP values can also be lower than expected in obese HF patients [28].

On the basis of these limitations, there has been growing interest in combining different biomarker measurements, reflecting multiple biological processes of HF progression, in order to improve risk stratification and overcome some of the limits of natriuretic peptides [29,30]. In the present study, the chosen biomarkers panel integrates the information provided by NT-proBNP with two fibrosis biomarkers, i.e., ST2 and Gal-3. ST2 is a member of the interleukin-1 receptor-like family of proteins expressed on fibroblasts and cardiomyocytes. It is has a transmembrane (ST2L) and a soluble circulating isoform (sST2). When sST2 binds interleukin-33, which is secreted in response to myocardial injury and inflammation, it acts as a decoy receptor preventing the anti-fibrotic and anti-hypertrophic signaling pathway of myocardial cells [31]. ST2 is also sensitive to mechanical stretch [32], similarly to natriuretic peptides, so it exhibits dynamic concentrations in the short-term in response to the load, decreasing rapidly after effective therapy [33] without the same caveats as NT-proBNP [34,35]. Gal-3 is a soluble β-galactoside-binding lectin secreted by activated macrophages. When released into the extracellular space it interact with surface receptors leading to signaling pathways for several cellular actions involving cell growth and differentiation, cell adhesion, apoptosis, and angiogenesis. At the myocardial level inflammatory processes and macrophage activation occurring in HF promote cardiac fibroblast proliferation, collagen deposition and, in turn, ventricular remodeling [36]. As opposed to ST2 and natriuretic peptides, Gal-3 has very stable levels and is considered a marker of the underlying fibrotic process that could provide further information besides that carried by the load-related biomarkers [37].

Our results confirm that Gal-3 and ST2 carry different information from NT-proBNP. This is particularly evident when the correlation with the other clinical variables evaluated in our study is considered. In fact, ST2 appears less influenced by age or renal function. Gal-3 seems to be a biomarker strongly influenced by renal function, weakly correlated with left ventricular diastolic and systolic functions.

Moreover, our study demonstrates that the integration of the information carried by altered Gal-3 and ST2 to that of NT-proBNP improves the risk stratification of CHF patients by adding an incremental prognostic value. In particular, it is possible to divide the population into four groups of risk according to the number of altered biomarkers, i.e., a group at low risk with no biomarker elevated, a group at low–intermediate risk with only one biomarker elevated (NT-proBNP or one of the fibrosis biomarkers), a group at intermediate–high risk with two biomarkers elevated, and a high risk group with all the biomarkers altered.

From a methodological point of view we demonstrated this hypothesis by prospectively studying a homogenous group of outpatients in stable clinical conditions, following-up on them for one year. We censored the follow-up at this time considering that any prognostic parameter evaluated at the time of a visit should provide information about short- and mid-term follow-up in order to better assess whether an intensive follow-up was needed or not. Moreover, we used the cut-offs of biomarkers currently recommended in order to better reflect the use of biomarkers in routine practice.

Finally, to better define the prognostic usefulness of the biomarker prognostic model proposed, we compared it with the BIOSTAT-CHF, a recently-validated model for predicting mortality and hospitalization of CHF patients. The biomarker score showed a similar C-index and proved to be significantly and independently associated with events, adding incremental prognostic information to the BIOSTAT-CHF model.

The main limitation of our study is that the real impact of a biomarker strategy should be demonstrated by studies evaluating if the management of patients based on the biomarkers-strategy is able to reduce heart failure hospitalization thus supporting their use by a cost-effectiveness analysis. Moreover, the biomarker strategy should be compared with that based on echocardiography in order to better understand if it can be alternative or not. Finally, the clinical characteristics of our population do not reflect those of the general population affected by heart failure. They were younger and mainly affected by heart failure with reduced ejection fraction. This is due to the fact that our heart failure unit is a regional tertiary center with a program for heart transplantation and/or left ventricular assist device implantation. This is a further limitation of our study.

## 5. Conclusions

Our results support the possible usefulness of the combination of NT-proBNP, ST2, and Galectin-3 in order to identify the patients with the highest risk of heart failure progression in the short and mid-term follow-up. This can be due to the fact that these biomarkers can integrate with each other, reflecting different pathophysiological substrates leading to worsening heart failure.

## Figures and Tables

**Table 1:** Baseline clinical characteristics of all patients and of patients divided according to biomarkers above the cut-off.

Clinical Characteristics		Number of Increased Biomarkers				*p*
	All Patients	Zero	One	Two	Three	
	*n:* 315	*n:* 81	*n:* 91	*n:* 92	*n:* 51	
Age (years)	64 ± 13	59 ± 13	64 ± 13 *	65 ± 13 *	71 ± 10 *^,^†^,^‡	<0.001
Males (%)	81	88	79	76	80	0.247
NYHA class	2.4 ± 0.6	2.0 ± 0.5	2.2 ± 0.5 *	2.6 ± 0.5 *^,^†	2.8 ± 0.5 *^,^†^,^‡	<0.001
MBP (mm Hg)	90 ± 11	94 ± 10	94 ± 11	88 ± 10 *^,^†	85 ± 9 *^,^†	<0.001
BMI (kg/m^2^)	27 ± 5	29 ± 5	29 ± 5	27 ± 5	27 ± 5	0.026
Atrial fibrillation (%)	20	4	15 *	30 *^,^†	35 *^,^†	<0.001
Ace-inhibitors/ARBs (%)	79	89	85	73 *^,^†	57 *†^,^‡	<0.001
Beta-blockers (%)	96	100	97	95	92	<0.001
Diuretics (%)	92	84	91 *	97 *	100 *^,^†	<0.001
Digoxin (%)	11	4	13	13	18	0.476
MRAs (%)	70	70	64	78 †	65 ‡	0.030
ICD (%)	87	91	82	87	86	0.549
CRT (%)	34	37	37	32	26	0.149
LVEF (%)	33 ± 9	37 ± 7	34 ± 8 *	31 ± 10 *	31 ± 11 *	<0.001
E/e’	14 ± 7	10 ± 4	13 ± 6	16 ± 8 *^,^†	17 ± 10 *^,^†	<0.001
TAPSE (mm)	19 ± 4	20 ± 4	20 ± 4	18 ± 4 *^,^†	17 ± 10 *^,^†	<0.001
MR (a.u.)	1.8 ± 0.9	1.4 ± 0.8	1.6 ± 0.8	2.0 ± 1.0 *^,^†	2.1 ± 0.9 *^,^†	<0.001
TR (a.u.)	1.8 ± 1.0	1.4 ± 0.8	1.5 ± 0.7	2.0 ± 1.1 *^,^†	2.4 ± 1.1 *^,^†^,^‡	<0.001
CVP (mmHg)	5 ± 4	3 ± 2	4 ± 3	6 ± 5 *^,^†	8 ± 5 *^,^†^,^‡	<0.001
PASP (mmHg)	37 ± 14	31 ± 11	33 ± 9	40 ± 12 *^,^†	48 ± 19 *^,^†^,^‡	<0.001
GFR-EPI (mL/min/1.73 m^2^)	71 ± 26	87 ± 20	75 ± 24 *	66 ± 25 *^,^†	48 ± 17 *^,^†^,^‡	<0.001
Sodium (mmol/L)	139 ± 8	139 ± 15	140 ± 3	139 ± 3	138 ± 5	0.726
Hb (g/dL)	13.5 ± 1.6	14.1 ± 1.2	13.8 ± 1.6	13.4 ± 1.7 *	12.4 ± 1.3 *^,^†^,^‡	<0.001
CRP (mg/L)	5.4 ± 7.7	4.0 ± 2.6	4.9 ± 6.1	6.4 ± 10.3	6.9 ± 9.6	0.084
NT-proBNP (pg/mL)	2294 ± 3642	376 ± 256	1127 ± 1007	3145 ± 2125 *^,^†	5888 ± 6625 *^,^†^,^‡	<0.001
ST2 (ng/mL)	40.70 ± 27.52	24.98 ± 5.73	35.94 ± 13.88 *	47.20 ± 27.16 *^,^†	62.47 ± 44.84 *^,^†^,^‡	<0.001
Galectin-3 (ng/mL)	16.0 ± 7.1	11.2 ± 2.9	13.6 ± 4.2 *	17.3 ± 6.7 *^,^†	25.7 ± 6.7 *^,^†^,^‡	<0.001

Mean values ± SD or percentage of patients. *p* test ANOVA for continuous variables; *p* test F-Fisher for categorical variables. * *p <* 0.05 vs. zero group; † vs. one group; ‡ vs. two group at Newman-Keuls post-hoc analysis. ARBs: angiotensin II receptor blockers; a.u., arbitrary units; BMI, body mass index; CRP, C-reactive protein; CRT, cardiac resynchronization therapy; CVP, central venous pressure; GFR-EPI, glomerular filtration rate by EPI formula; Hb, hemoglobin; ICD, implantable cardioverter defibrillator; LVEF, left ventricular ejection fraction; MBP, mean blood pressure; MR, mitral regurgitation; MRA, mineral corticoid receptor antagonists; NYHA, New York Heart Association; NT-proBNP: brain natriuretic peptide; PASP, systolic peak of pulmonary arterial pressure; RRI, renal resistance index; TAPSE, peak of tricuspid annular plane systolic excursion; TR, tricuspid regurgitation.

**Table 2:** Clinical correlates of biomarkers.

Variables	Age	NYHA	BMI	LVESV	LVEF	E/e’	MR	TR	PAPS	CVP	TAPSE	HB	GFR-EPI
NT-proBNP	***r*: 0.160**	***r*: 0.399**	*r*: −0.102	***r*: 0.297**	***r*: −0.350**	***r*: 0.326**	***r*: 0.335**	***r*: 0.244**	***r*: 0.506**	***r*: 0.347**	***r*: −0.244**	***r*: −0.315**	***r*: −0.376**
	***p*: 0.005**	***p <* 0.001**	*p*: 0.071	***p <* 0.001**	***p <* 0.001**	***p <* 0.001**	***p <* 0.001**	***p <* 0.001**	***p <* 0.001**	***p <* 0.001**	***p <* 0.001**	***p <* 0.001**	***p <* 0.001**
ST2	*r*: 0.057	***r*: 0.223**	***r*: −0.121**	*r*: 0.016	***r*: −0.145**	*r*: 0.114	***r*: 0.146**	***r*: 0.146**	***r*: 0.336**	***r*: 0.333**	***r*: −0.146**	***r*: −0.189**	***r*:−0.125**
	*p*: 0.314	***p <* 0.001**	***p*: 0.032**	*p*: 0.787	***p <* 0.001**	*p*: 0.058	***p*: 0.010**	***p*: 0.011**	***p <* 0.001**	***p <* 0.001**	***p*: 0.011**	***p*: 0.001**	***p*: 0.027**
Galectin-3	***r*: 0.344**	***r*: 0.333**	*r*: −0.060	*r*: 0.071	***r*: −0.114**	***r*: 0.315**	***r*: 0.191**	***r*: 0.251**	***r*: 0.2910**	***r*: 0.252**	***r*: −0.161**	***r*: −0.313**	***r*: −0.646**
	***p <* 0.001**	***p <* 0.001**	*p*: 0.287	*p*: 0.223	***p*: 0.044**	***p <* 0.001**	***p*: 0.001**	***p <* 0.001**	***p <* 0.001**	***p <* 0.001**	***p*: 0.005**	***p <* 0.001**	***p <* 0.001**

Statistically significant correlations are in bold, for abbreviations see Table 1.

**Table 3:** Predictive value of high NT-proBNP, galectin-3, and ST2 serum levels.

Variables	Univariate Cox Regression Analysis			Multivariate Cox Regression Analysis		
	HR (95% CI)	*p*	C-index	HR (95% CI)	*p*	C-index
NT-proBNP > 1000	7.94 (3.78–16.67)	<0.001	0.71	5.15 (2.39–11.08)	<0.001	
ST2 > 34	4.06 (2.28–7.24)	<0.001	0.66	2.95 (1.64–5.25)	<0.001	0.79
Galectin-3 > 17.9	3.18 (2.01–5.48)	<0.001	0.64	2.04 (1.22–3.41)	0.007	

**Table 4:** Predictive value of the score based on the presence and the number of high NT-proBNP, galectin-3, or ST2 serum levels.

Variables	Univariate Cox Regression Analysis						
	HR (95% CI)	*p*	C-index				
**Biomarker score**	2.96 (2.21–3.95)	<0.001	0.78				
							
	**Multivariate Cox Regression Analysis**						
	**HR (95% CI)**	***p***	**C-index**	**IDI**	**(*p*)**	**Free NRI**	
Biomarker score added to reference model *	1.52 (1.06–2.17)	0.023	0.87	0.026	0.031	0.398	<0.001
							
**Biomarker score added to traditional biomarkers †**	2.64 (1.90–3.67)	<0.001	0.80	0.109	<0.001	0.849	<0.001
							
**Biomarker score added to BIOSTAT-CHF**	2.49 (1.84–3.36)	<0.001	0.80	0.118	<0.001	0.43	<0.001
							
**Biomarker score added to magic**	1.89 (1.35–2.65)	<0.001	0.83	0.777	<0.001	0.589	<0.001

* After correction for a reference model (i.e., beta blocker therapy, haemoglobin < 12 g/dL, LVEF < 30%, NYHA class 3, at least moderate tricuspid regurgitation). † After correction for GFR < 60 mL/kg/min × 1.73 m^2^, urea > 50 mg/dL, Na < 135 mEq/L, and hemoglobin < 12 g/dL.
